# A Female-Emitted Pheromone Component Is Associated with Reduced Male Courtship in the Parasitoid Wasp *Spalangia endius*

**DOI:** 10.1371/journal.pone.0082010

**Published:** 2013-11-20

**Authors:** Sophie L. Mowles, Bethia H. King, Robert S. T. Linforth, Ian C. W. Hardy

**Affiliations:** 1 School of Biosciences, University of Nottingham, Loughborough, Leicestershire, United Kingdom; 2 Department of Biological Sciences, Northern Illinois University, DeKalb, Illinois, United States of America; University of Melbourne, Australia

## Abstract

During courtship interactions, the courted individual may not always be prepared to mate. For example, mating or courtship may be detrimental to its fitness and resistance is expected under these circumstances. As such, various resistance strategies have evolved, from physically fending off courting individuals to producing behavioural signals of unreceptivity. In the parasitoid wasp *Spalangia endius*, females rarely re-mate and mated females are avoided by males in favour of virgin females. Further, mated females appear to advertise their mating status by the release of a pheromone component (methyl 6-methylsalicylate), but direct evidence of the nature of this release is lacking. Here we used real-time chemical analysis to track the emission of the pheromone component during courtship interactions between virgin males and either virgin or mated females. We found that females actively release methyl 6-methylsalicylate when courted and that significantly greater concentrations are released by previously mated females. Further, high concentrations of this component are associated with both the prevention and termination of courtship.

## Introduction

During courtship interactions, the courter (typically the male) attempts to persuade the courted individual (typically the female) to mate, using various displays of quality [1]. Sometimes, however, attending to courtship or accepting a mating is not in the best interests of the female, and resistance is expected under these circumstances. Resisting the courtship and mating attempts of males may allow females to avoid: injury [2], sexually transmitted diseases [3], predation risk [4,5] or harassment that may reduce opportunities to feed and oviposit [6-8] or even result in the inadvertent death of the female [9-11].

Such male-female disparities in the fitness consequences of mating have resulted in sexual conflict and the evolution of a variety of resistance strategies by females [12-14]. These resistance strategies vary in their level of hostility towards the male, with some females physically rebuffing courting males [15,16], while others simply move to areas where they can avoid courtship encounters [6,7]. These strategies can be costly, involving energetic expenditure by the resisting female and the potential for injury during the struggle [17]. Another strategy is to produce signals of unreceptivity, which include not only physical behaviour but also the use of pheromones (e.g. in the garter snake, *Thamnophis sirtalis* [18], and in some insects [19]), but most of these cases are due to a previous male depositing pheromones on the surface of the female’s body (e.g. 20). It remains to be seen, however, how female-produced chemical signals of resistance are associated with the behaviour of courting males. 

**Figure 1:**
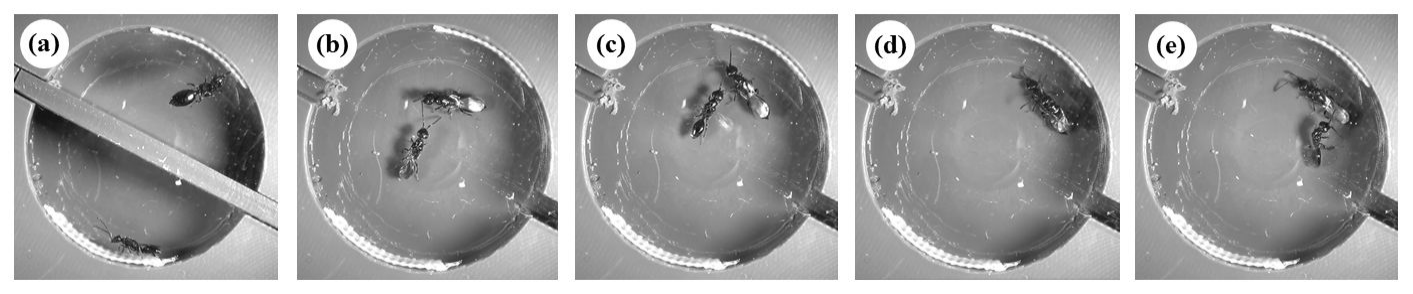
A typical courtship interaction between a virgin male (bottom compartment) and a previously mated female (top compartment) *Spalangia endius*. (a) The wasps start either side of the barrier during the ‘pre-interaction’ period, (b) the male approaches the female during the ‘pre-courtship period’, (c) wing-fans, (d) mounts the female and (e) retreats following pheromone release.

 In the parasitoid wasp *Spalangia endius*, females rarely mate more than once, even if repeatedly courted [21,22]. Males approach and ‘wing-fan’ almost every female (see Figure 1 and supporting Video 1), a courtship behaviour whereby they move their wings rapidly up and down. If courtship proceeds to mounting, the male begins a vibratory behaviour where he performs rapid push-ups of his whole body on the female’s dorsum [23,24], which then typically leads to copulation. In interactions with previously mated females, however, males regularly terminate their courtship attempts at this stage or prior to mounting [25]. The retreat of males from mated females does not appear to be in response to visible aggression by the female, suggesting that females may be releasing a chemical volatile. Female *S. endius* are known to produce the pheromone component, methyl 6-methylsalicylate, which when isolated in laboratory studies, caused males to either respond by behavioural arrestment, or by wing-fanning (courtship) [26]. By coupling behavioural observations of interacting wasps with real-time monitoring of pheromone release, we identify the timing and concentration of pheromone released, whether it differs between virgin and mated females, and record the associated behaviour of the courting male. 

## Materials and Methods

### Ethics statement

All animal work was carried out in accordance with the ASAB/ABS Ethical Guidelines. Approval was not necessary for this work as it involves insects, which require no research permits or approval in the UK.

### Study organisms

*Spalangia endius* Walker (Hymenoptera: Pteromalidae) were obtained from a well-studied strain [21-27] that was originally collected from Florida, USA. They were maintained at 25°C under a 12/12 h light/dark light regime and were raised using a natural host, pupae of the housefly, *Musca domestica* L. (Diptera: Muscidae) [26]. To obtain experimental animals, the parasitised pupae were placed individually in test tubes sealed with a small wad of cotton wool. As this species is solitary (only one wasp emerges from each pupa), this procedure ensured that the wasps that emerged remained virgin prior to the trials.

Each newly emerged wasp was sexed using a binocular microscope. One group of wasps (25 females and 25 males) were placed as pairs into ‘mixed sex’ test tubes, each containing one male and one female. A second group (25 females and 50 males) were placed individually into ‘single sex’ test tubes in order to retain virgins. The individuals in the mixed sex test tubes were observed to ensure that mating had taken place, after which the males were removed from the experiment and the mated females retained. Following an acclimation period of 3 h, wasps were assigned to pairs comprising either: i) a virgin female and virgin male or ii) a mated female and virgin male. Each pair was then placed into a 10 mm diameter PVC interaction chamber covered by a clear Perspex lid, one wasp on either side of an opaque barrier (Figure 1a), and allowed to acclimate for a further 30 min to ensure that any pheromone emissions from the female were associated with interacting with the male, rather than with being placed in a new container.

### Real-time pheromone analysis and behavioural observations

The real-time release of pheromones during the interaction was monitored by an Atmospheric Pressure Chemical Ionisation Mass-Spectrometer (APCI-MS) [28]. In preliminary trials, using different individual wasps, both females and males were stressed in front of the APCI-MS intake in full scan mode by gently pressing each wasp against the side of a test tube using a paintbrush. This allowed us to confirm what volatiles were released. Females released a compound of molecular weight 166, known from previous work to be methyl 6-methylsalicylate [26], whilst the males did not. The male wasps did, however, release a different compound of molecular weight 196 when stressed. Each interaction was hence monitored using the APCI-MS in selected ion mode to detect the emission of methyl 6-methylsalicylate from the female wasps and the emission of the unknown compound (molecular weight 196) from the male wasps. 

Prior to the trials, the system was calibrated by first aspirating a gas phase sample of methyl-salicylate at 1.358 mg/m^3^ into the APCI-MS intake. This concentration (200p/bbv) is equivalent to 1.492 mg/m^3^ of methyl 6-methylsalicylate. Each wasp interaction block included a 3.5 mm diameter hole in the chamber wall, which allowed the intake of the APCI-MS sampling line to be inserted, drawing air out of the chamber at 25 ml/min. A further hole was placed in the opposite wall of the chamber to ensure that air flowed through the chamber, preventing the build-up of pheromones in the arena. Both holes were covered with a fine fabric mesh to prevent the escape of the wasps.

At the start of each trial, the interaction block was connected to the intake of the APCI-MS by the section containing the female in order to record any pheromone emissions produced by the female prior to interacting with the male. After a ‘Pre-Interaction’ period of approximately 1 min, the barrier filling the slot was withdrawn, allowing the wasps to interact (Figure 1b). Each interaction was recorded from above using a Sony HDRXR160EB Handycam Camcorder until courtship had taken place or until a 10 minute period had elapsed, after which it was deemed that courtship was unlikely to occur. Behavioural data were scored from the digital recordings using JWatcher version 1.0 event recording software [29]. Each interaction was scored for the occurrence and duration of the following courtship behaviours: the rapid wing-fanning directed at the female, the male mounting the female, the vibratory push-ups performed while situated on the female’s dorsum, and successful copulations (see 23,24). The video data were then combined with the real-time spectra obtained from the APCI-MS for analysis. Data on amount of chemical released were analysed using StatView 5.0 (SAS, Cary, NC) and Genstat 14.0 was used for logistic analysis [30]. Where necessary, data that were not normally distributed were log_10_-transformed before analysis in order to meet the requirements of the parametric tests. 

## Results

### Frequency of matings

Significantly more virgin females than previously mated females copulated during the courtship trials (chi-squared test of association: χ^2^ = 23.269, d.f. = 1, *P* < 0.0001), with 20 of the 25 virgin wasps mating compared to 3 of the 25 mated wasps re-mating.

### Timing of pheromone emissions

**Figure 2:**
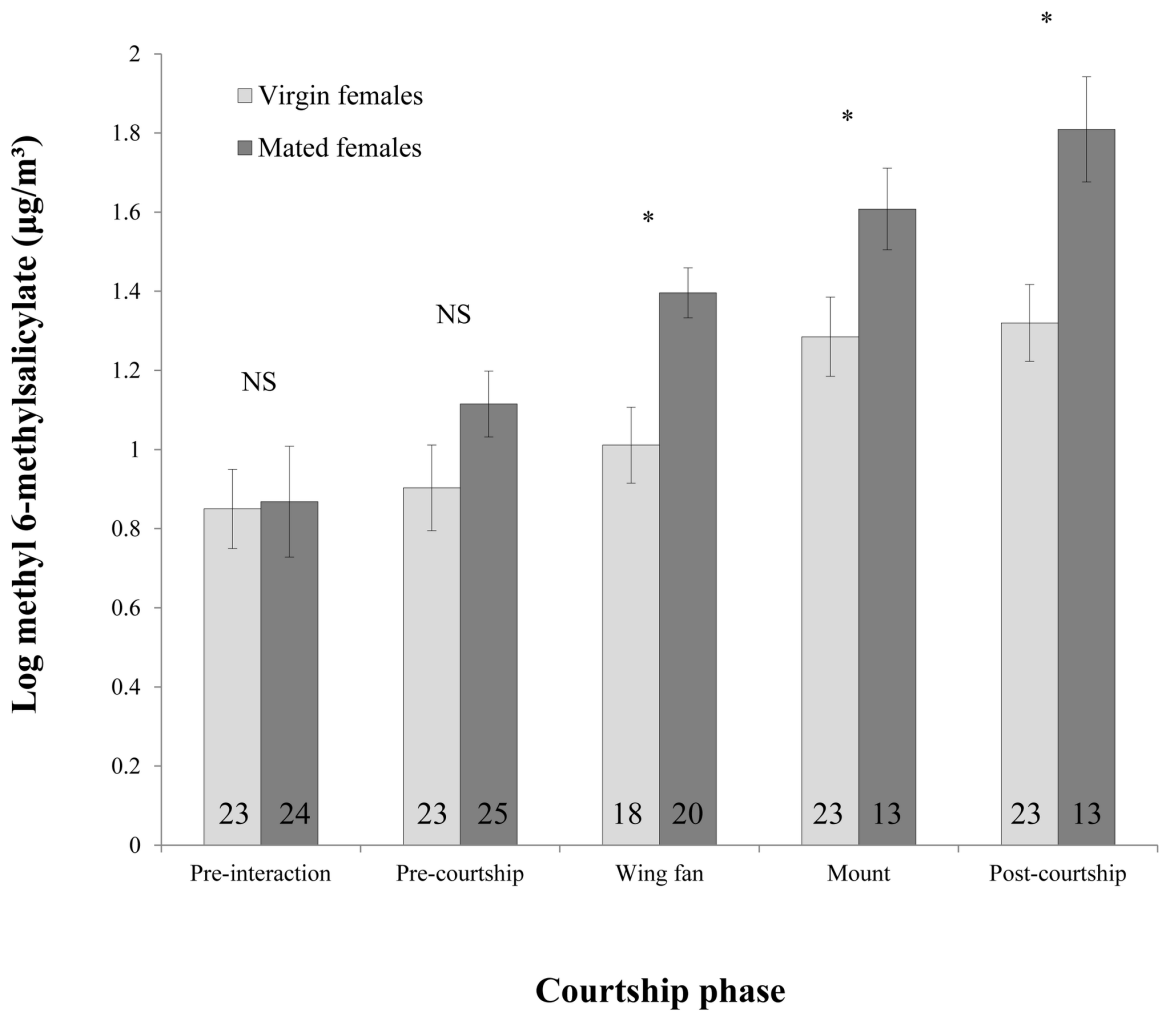
The increase in methyl 6-methylsalicilate over the course of a courtship interaction, which has been divided into five discrete behavioural phases for clarity. Error bars show standard errors. Asterisks indicate significant differences within phases. As not all wasps progressed to each stage of courtship, numbers indicate sample sizes.

No volatile compounds were released by the male wasps during the behavioural trials and thus we focus on female emissions only. To determine whether the amount and timing of methyl 6-methylsalicylate release differed between mated females and virgin females, we carried out a repeated measures ANOVA that incorporated the different phases of the courtship interaction. The between subject factor was female mating status (virgin or mated), the response variable was the mean concentration of methyl 6-methylsalicylate and the repeated measure was the courtship phase: pre-interaction (prior to barrier withdrawal; Figure 1a), pre-courtship (prior to any courtship behaviour by the male; Figure 1b), during the ‘wing-fanning’ period (Figure 1c), during mounting (Figure 1d), and post-courtship (the 30 seconds immediately following male dismount; Figure 1e). The mean concentration of methyl 6-methylsalicilate differed significantly between the courtship phases (*F*_4,108_ = 12.868, *P* < 0.0001; Figure 2) and was also significantly higher in the mated female treatment than in the virgin female treatment (*F*_1,108_ = 12.803, *P* = 0.0013; Figure 2). There was no significant overall interaction between the female’s mating status and the phase of courtship (*F*_4,108_ = 0.556, *P* = 0.6953; Figure 2). As repeated measures analysis does not allow for the application of post hoc tests within ANOVA, and due to diminishing sample sizes within each phase of the encounter (see Figure 2), a series of one-factor ANOVAs was used to examine the effect of female mating status on the concentration of methyl 6-methylsalicilate released during each of the five courtship phases. There was no difference in the mean concentration of methyl 6-methylsalicilate between virgin females and mated females during the pre-interaction period (*F*_1,48_ = 0.088, *P* = 0.7683) or during the pre-courtship period (*F*_1,46_ = 2.468, *P* = 0.1231), but methyl 6-methylsalicilate was significantly higher in the mated female treatment during wing-fanning (*F*_1,36_ = 11.748, *P* = 0.0015), mounting (*F*_1,34_ = 4.347, *P* = 0.0446) and post-courtship (*F*_1,34_ = 8.939, *P* = 0.0052; Figure 2).

### Pheromone emissions and male courtship behaviour

In order to evaluate whether the emission of methyl 6-methylsalicilate is related to male courtship behaviour, we carried out a series of Pearson product-moment correlations between the maximum concentration of methyl 6-methylsalicilate and i) the latency of the male to perform courtship behaviours and ii) the duration of courtship performed by the male. If the male did not perform these behaviours, they were excluded from the analysis.

**Figure 3:**
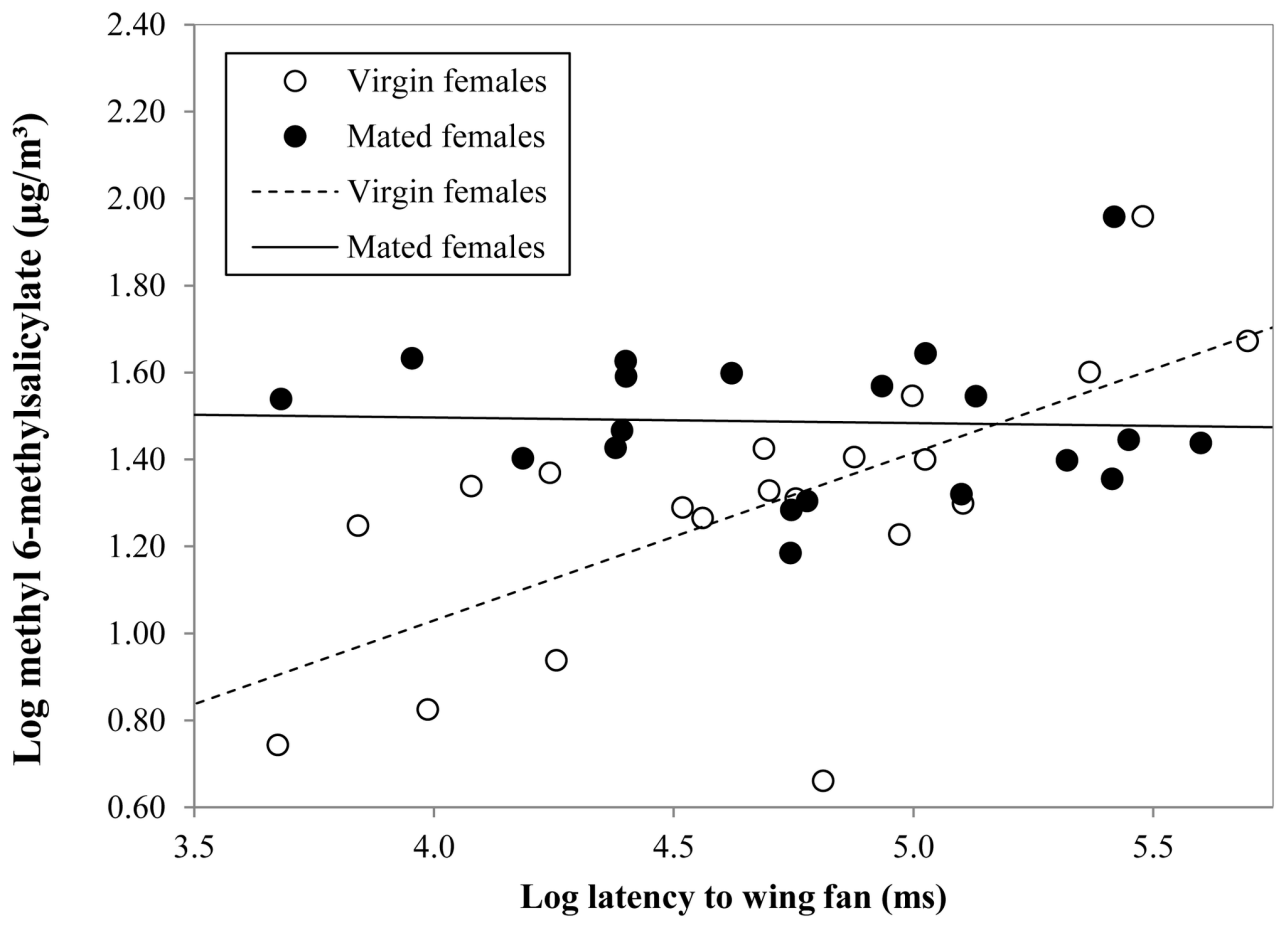
Bivariate scatterplot with fitted regression line illustrating the positive relationship in the virgin female treatment between the maximum pre-courtship concentrations of methyl 6-methylsalicilate and the latency for the male to initiate the wing-fanning courtship behaviour. The fitted regression line, with a slope of zero, for the mated female treatment indicates the lack of relationship.

The latency for the male to initiate wing-fanning was positively correlated with the maximum concentration of methyl 6-methylsalicilate emitted by the female during the pre-courtship period (*r* = 0.414, df = 38, *P* = 0.0073). When the two treatment groups were analysed separately, this relationship was present in the virgin female group, but was absent in the mated female group (Table 1; Figure 3). 

**Table 1:** Pearson product-moment correlations of latency to wing fan against maximum methyl 6-methylsalicylate emission during the pre-courtship period.

	*r*	df	*P*
All individuals	0.414	38	0.0073
Virgin female treatment only	0.674	18	0.0007
Mated female treatment only	-0.040	18	0.8691

**Figure 4:**
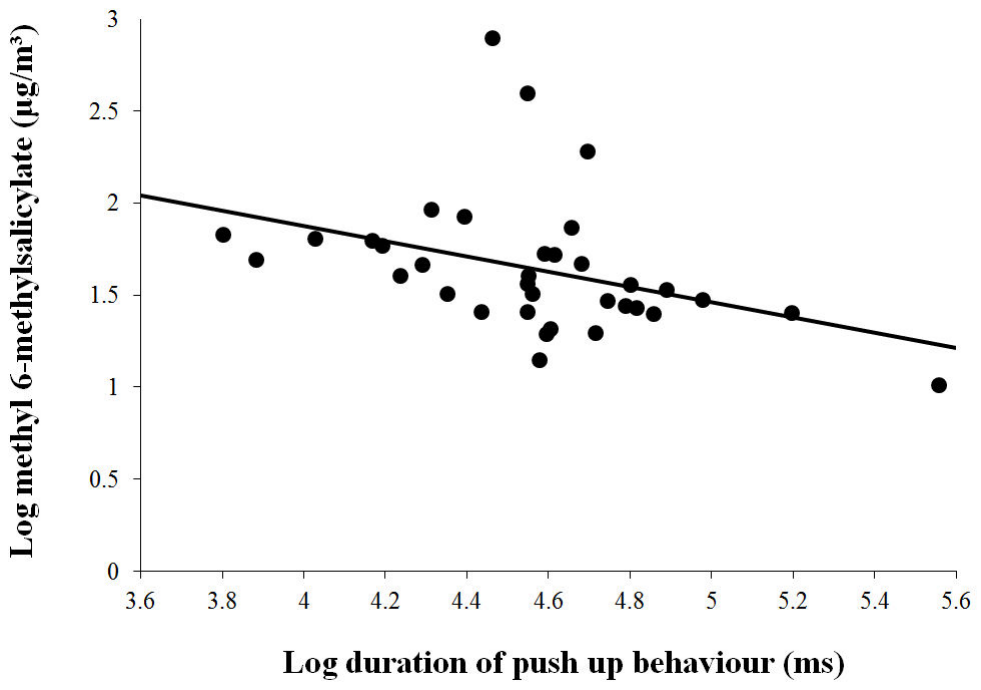
Bivariate scatterplot with fitted regression line illustrating the negative relationship between the maximum concentrations of methyl 6-methylsalicilate emitted while the male is mounted on the female and the duration of the vibratory courtship ‘push-up’ behaviour produced by the male.

The total time that the male spent performing vibratory push-ups was negatively correlated with the maximum concentration of methyl 6-methylsalicilate during: the pre-courtship period (*r* = -0.375, df = 33, *P* = 0.0256), wing-fanning (*r* = -0.564, df = 28, *P* = 0.0009), and during mounting (*r* = -0.382, df = 33, *P* = 0.0227; Figure 4). When the two treatment groups were analysed separately, a negative correlation remained between the total time that the male spent performing vibratory push-ups and the maximum concentration of methyl 6-methylsalicilate emitted by mated females, while all other relationships were non-significant (Table 2).

**Table 2:** Pearson product-moment correlations of the total duration of push-ups against maximum methyl 6-methylsalicylate emission during: a) the pre-courtship period, b) wing-fanning, and c) the period mounted.

	*r*	df	*P*
**a) pre-courtship period**			
All individuals	-0.375	33	0.0256
Virgin female treatment only	-0.183	20	0.4198
Mated female treatment only	-0.642	11	0.0161
**b) wing-fanning**			
All individuals	-0.564	28	0.0009
Virgin female treatment only	-0.333	17	0.1658
Mated female treatment only	-0.239	9	0.4899
**c) the period mounted**			
All individuals	-0.382	33	0.0227
Virgin female treatment only	-0.359	20	0.1015
Mated female treatment only	-0.009	11	0.9777

### Pheromone emissions and likelihood of mounting

**Figure 5:**
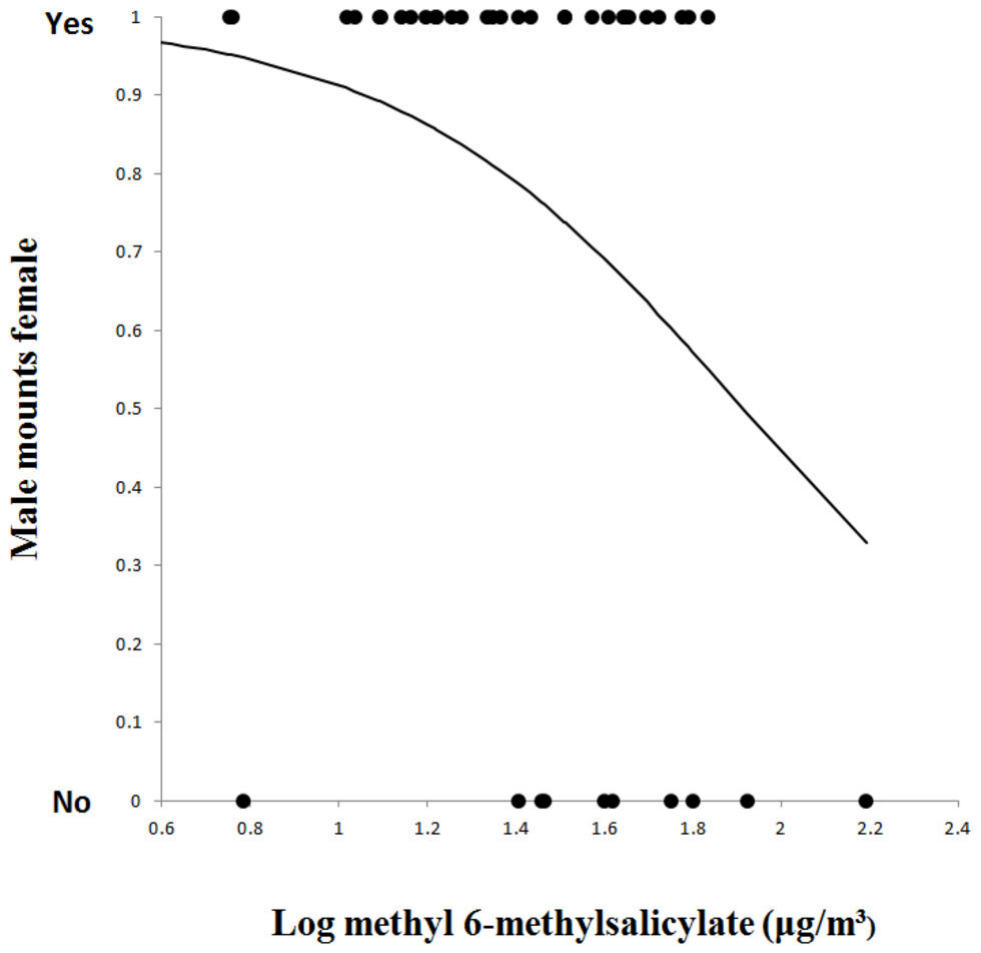
Logistic regression illustrating the decreasing probability of the male going on to mount the female as the maximum concentration of pheromone emitted during the wing-fanning period increases.

We used logistic regression [31] to assess whether the concentration of the pheromone produced by females affected the likelihood of the male mounting the female. The explanatory variable was the maximum concentration of the pheromone produced by all females (mated and virgin) during the wing-fanning period (immediately before the decision to mount and perform vibratory push-ups) while the response variable was whether or not mounting occurred. Males were significantly less likely to go on to mount the female when pheromone concentrations were higher (G_1_ = 4.13, *P* = 0.042; Figure 5).

## Discussion

We have found that both virgin and mated female *Spalangia endius* wasps emit the pheromone component methyl 6-methylsalicilate and that its concentration rises throughout courtship. Females that have previously mated, however, produce significantly more of the pheromone component than do virgin females. Further, the mean concentration of the pheromone component released by virgin and mated females is similar at the beginning of interactions, but the difference in emission between virgin and mated females becomes more pronounced as courtship progresses. This indicates that increased emission by previously mated females is likely in response to courtship attempts by the males.

The emission of high levels of methyl 6-methylsalicilate appears to be related to the inhibition of courtship attempts of males at a variety of stages: high initial levels may postpone wing-fanning, high levels during wing-fanning may reduce the likelihood of the male proceeding to mount the female and high levels during the mounted period may shorten the time that the male spends performing the courtship push-up behaviour. Thus, the emission of high concentrations of methyl 6-methylsalicilate by females may postpone, prevent and shorten courtship attempts by males. In contrast, in previous studies involving chemical trials, methyl 6-methylsalicilate has also been shown to stimulate the wing-fanning courtship behaviour in males [25,26]. Thus, methyl 6-methylsalicilate can act as an aphrodisiac pheromone, attracting males and stimulating courtship. However, here we have shown that previously mated females actively release methyl 6-methylsalicilate at higher concentrations in response to superfluous courtship, whereupon it appears to act as an ‘anti-aphrodisiac’, associated with the termination of courtship attempts. There is the alternative possibility that males have evolved a strategy that results in the increased production of methyl 6-methylsalicilate from females that they have mated with, perhaps through the biochemical actions of seminal fluid components [32]. This process would be adaptive from the male’s perspective, releasing him from some sperm-competition by rendering females monandrous. In the present study, however, some males attempted to mount the females without first wing-fanning. This process appeared to startle the females and was associated with the prompt emission of high levels of methyl 6-methylsalicilate, even by virgin females. This demonstrates that the emission of high levels of methyl 6-methylsalicilate is the result of a behavioural decision by the female to reject the current male.

The production of any pheromone is a costly process [33-36] and performing an energetically expensive behaviour would suggest that the costs incurred to females in the act of resistance are less than those of re-mating. Thus, the costs of re-mating, of mating, or of simply being exposed to courtship are likely to be high for female *S. endius*. It is unlikely that internal injuries cause female reluctance to re-mate as *S. endius* lack the armoured genitalia seen in other insect species such as the flies *Drosophila bipectinata* [37] and *Sepsis cynipsea* [38] and beetles of the genus *Callosobruchus* [39]. Further, as female *S. endius* must open their genital orifices in order to mate [23], forced copulations are unlikely in this species. Thus, female *S. endius* may not be responding adversely to the prospect of re-mating but to the prospect of sexual harassment by superfluous courtship. This conclusion is supported by the increase in the emission of methyl 6-methylsalicilate throughout courtship in the virgin female treatment: the female may use elevated emission to influence the duration of courtship that she receives.

During courtship in *S. endius*, the male approaches the female, performing a wing-fanning display, after which he mounts the female and performs a vibratory push-up behaviour whilst situated on her dorsum. Although females subsequently burrow through carrion, manure, or rotting vegetation in search of hosts in which to oviposit [24], mating takes place on the surface, where such obvious courtship behaviours may also be apparent to predators. Further, as the courtship behaviour of *S. endius* involves the male remaining mounted on the female, his presence may interfere with the ability of the female to burrow for hosts [23], impeding oviposition (e.g. 6). By resisting male courtship, it seems likely that female *S. endius* can reduce the costs associated with sexual harassment by males; and any physiological cost of increased pheromone production may be less than the cost of failing to oviposit.

The use of an ‘attractive’ sex pheromone as an ‘anti-aphrodisiac’ is an unusual change in function and the mechanism by which it is associated with the cessation of male courtship may be as a signal of unreceptivity, or as a weapon (see 28). As females rarely re-mate, the emission of high concentrations of methyl 6-methylsalicilate may indicate to the courting male that the female is unreceptive and unlikely to mate, thus acting as a signal. However, some males persist for longer than others and some females do indeed go on to re-mate. Further, as the concentration of the pheromone is correlated with the probability of the male withdrawing from a courtship interaction, it seems likely that the pheromone is acting as more than a signal of unreceptivity and that there are consequences for males that are exposed to it. It appears that elevated levels of methyl 6-methylsalicailate may function as a nociceptive stimulus. In a laboratory study using extracts of methyl 6-methylsalicilate, the antennae of males were shown to be sensitive to the compound and males responded behaviourally to it by arrestment [26]. Further, previous studies have demonstrated that males exhibit decreased sexual responsiveness after mating [23] and after interacting with mated females [24]. The decreased sexual responsiveness, even during future interactions with virgin females [22,24], suggests that being exposed to the pheromone has a direct effect on male behaviour beyond that of simply responding to a signal.

## Conclusions

There are several reasons that females may resist the courtship and mating attempts of males: to avoid injury, contracting sexually transmitted diseases, exposure to predation risk, or harassment that may reduce opportunities to feed and oviposit. Resistance strategies vary in their hostility and costliness, with some females physically rebuffing courting males while others simply move to areas where they can avoid courtship encounters. Here we have identified that a change in concentration of a pheromone component is associated with a change in function. Thus, female *S. endius* appear to have developed a strategy whereby they use an otherwise attractant sex pheromone as a deterrent by increasing the concentration at which it is released, thereby turning it into an ‘anti-aphrodisiac’. This mechanism may be evolutionarily important, not only for *S. endius*, but also for other species. That a change in concentration changes the function of a sex pheromone in *S. endius* is a novel mechanism that has remained untested in any taxa until now. Such concentration-mediated shifts in function may thus be more widespread than previously thought. Further, the process of increasing the concentration of a sex-attractant to render it an anti-aphrodisiac may have important evolutionary consequences as females do not need to produce a novel compound to deter superfluous courtship. This mechanism may thus facilitate the evolution of anti-aphrodisiac strategies. 

## Supporting Information

Video S1 **A typical courtship interaction between a virgin male (bottom compartment) and a previously mated female (top compartment) *Spalangia endius*.** The male approaches the female and proceeds to wing-fan, after which he mounts the female and performs rapid push-ups of his whole body on the female’s dorsum. The male then retreats following heightened pheromone release, without having mated with the female.
(MP4)
